# Super effect or methodological flaw: questioning the glucagon-like peptide-1 receptor agonist cancer narrative

**DOI:** 10.1007/s00125-025-06538-9

**Published:** 2025-09-08

**Authors:** Gregor A. Maier, Wolfgang Rathmann, Oliver Kuß

**Affiliations:** 1 Institute for Biometrics and Epidemiology, German Diabetes Center, Leibniz Center for Diabetes Research at Heinrich Heine University, Düsseldorf, Germany; 2 German Center for Diabetes Research, Partner Düsseldorf, München-Neuherberg, Germany; 3 Centre for Health and Society, Medical Faculty and University Hospital Düsseldorf, Heinrich Heine University, Düsseldorf, Germany

**Keywords:** Cancer, Confounding, Confounding by indication, Diabetes mellitus, Glucagon-like peptide-1 receptor agonist, Insulin, Time-lag bias

*To the Editor:* We read with interest the recently published paper entitled ‘Glucagon-like peptide-1 receptor agonists and gastrointestinal cancer risk in individuals with type 2 diabetes’ by Kuo et al [1]. The authors investigated the effect of glucagon-like peptide-1 receptor agonists (GLP-1RAs) compared with other glucose-lowering drugs in people with type 2 diabetes from the US TriNetX Research Network. The primary outcome was the incidence of obesity-related gastrointestinal (GI) cancers, comprising oesophageal, gastric, pancreatic, gallbladder, biliary and colorectal cancer. Each of these GI cancer types was also assessed separately as a secondary outcome.

In particular, the effect of GLP-1RAs compared with insulin was striking, with consistently strong protective effects across all outcomes. The primary outcome showed a 71% reduction with GLP-1RAs vs insulin (adjusted HR [aHR] 0.29; 95% CI 0.23, 0.37), and secondary outcomes ranged from a 62% reduction for oesophageal cancer (aHR 0.38; 95% CI 0.19, 0.80) to even more extreme effects such as an 87% reduction for biliary/gallbladder cancer (aHR 0.13; 95% CI 0.05, 0.37) vs insulin [1]. These extreme effects persisted in analyses stratified by BMI, age and sex, as well as in sensitivity analysis that included people who switched to other glucose-lowering drugs.

While at first sight such consistent findings might appear to strengthen the authors’ findings, we feel that they are more likely due to methodological weaknesses and not to a causal protective effect of GLP-1RAs. For example, we believe that the potential benefits of GLP-1RAs for cancer risk reduction reported previously in the study by Wang et al [2] may be due to confounding by indication [3], which is a form of confounding that arises when the medical condition that led to treatment is itself an independent risk factor for the outcome [4].

We agree with the authors’ use of a new-user study design in their non-randomised setting. However, we feel that the comparison with insulin does not fulfil the criteria of an active comparator. The primary advantage of a well-chosen active comparator is its ability to reduce confounding by indication by comparing therapeutically similar or exchangeable drugs prescribed to people who are in clinical equipoise [4]. This assumption does not hold true for the chosen comparison. Considering the progressive course of type 2 diabetes and the relationship between worsening of disease and treatment escalation, duration of diabetes plays a key role in the development of disease-specific outcomes (including cancer) [5]. Insulin initiation typically represents treatment failure (e.g. HbA_1c_ target not achieved) of other glucose-lowering drugs and occurs later in the course of type 2 diabetes than the initiation of GLP-1RAs, which are commonly used as second- or third-line therapy [6]. This difference in timing of drug prescriptions introduces the potential for time-lag bias, as comparisons involve people at different timepoints in the course of type 2 diabetes.

The authors acknowledge that they were not able to account for diabetes duration [1]. However, we feel that this limitation most likely explains much of the observed large protective effects of GLP-1RAs. In line with this hypothesis, no dramatic reductions in the aHR for GI cancer risk were found with GLP-1RAs compared with other second- or third-line drugs such as sodium–glucose cotransporter 2 (SGLT2) inhibitors (aHR 0.80; 95% CI 0.68, 0.96) [1]. As large differences in diabetes duration between these two groups are unlikely, the results are more plausible and less likely to be affected by time-lag bias.

Another strong indication of time-lag bias is evident in electronic supplementary material (ESM) Fig. 1a (Kaplan–Meier event probability curve for primary outcomes in all individual cohorts) in Kuo et al [1]. It is striking that the curves diverge immediately following the 1 year lag period after drug initiation. The immediate separation of both curves strongly suggests that both groups differed fundamentally in their baseline risk for GI cancer diagnoses before initiating the drugs. This is further supported by the fact that time from cancer initiation to clinical diagnosis typically spans multiple years for many types of cancer (e.g. 25.2 years for gallbladder cancer) [7]. Given these long periods, any cancers diagnosed shortly after drug initiation (here, directly after the 1 year lag period) cannot plausibly be attributed to the drugs themselves, which further undermines a causal interpretation of the observed protective effect. Finally, while the authors censored people who switched to other glucose-lowering drugs, they were not able to account for drug adherence [1]. Generally, intention-to-treat analyses dilute treatment effects compared with as-treated approaches, which consider actual drug exposure. Thus, by accounting for drug adherence, the effect of GLP-1RAs on the incidence of GI cancer would probably have been even stronger.

In conclusion, we would like to caution against interpreting the results of Kuo et al [1] as strong evidence of a ‘super effect’ of GLP-1RAs on cancer prevention, as indicated in the authors’ conclusions. This view is supported by historical debates regarding metformin and cancer prevention [8], which should have led to increased vigilance against premature conclusions of large protective effects of diabetes treatments. Instead, this study once more highlights the considerable challenges of time-related biases in pharmacoepidemiological research.

## Abbreviations

aHR: Adjusted HR
GI: Gastrointestinal
GLP-1RA: Glucagon-like peptide-1 receptor agonist
